# The optimal timing of elective surgery in sigmoid diverticular disease: a meta-analysis

**DOI:** 10.1007/s00423-022-02698-z

**Published:** 2022-10-10

**Authors:** Sascha Vaghiri, Dimitrios Prassas, Wolfram Trudo Knoefel, Andreas Krieg

**Affiliations:** Department of Surgery (A), Heinrich-Heine-University and University Hospital Duesseldorf, Moorenstr. 5, Bldg. 12.46, 40225 Duesseldorf, Germany

**Keywords:** Sigmoid diverticulitis, Minimally invasive sigmoidectomy, Timing of resection, Complication rates

## Abstract

**Purpose:**

The aim of this meta-analysis was to investigate the optimal time point of elective sigmoidectomy regarding the intraoperative and postoperative course in diverticular disease.

**Methods:**

A comprehensive literature research was conducted for studies comparing the operative outcome of early elective (EE) versus delayed elective (DE) minimally invasive sigmoidectomy in patients with acute or recurrent diverticular disease. Subsequently, data from eligible studies were extracted, qualitatively assessed, and entered into a meta-analysis. By using random effect models, the pooled hazard ratio of outcomes of interest was calculated.

**Results:**

Eleven observational studies with a total of 2096 patients were included (EE group *n* = 828, DE group *n* = 1268). Early elective sigmoidectomy was associated with a significantly higher conversion rate as the primary outcome in comparison to the delayed elective group (OR 2.48, 95% CI 1.5427–4.0019, *p* = 0.0002). Of the secondary outcomes analyzed only operative time (SMD 0.14, 95% CI 0.0020–0.2701, *p* = 0.0466) and time of first postoperative bowel movement (SMD 0.57, 95% CI 0.1202–1.0233, *p* = 0.0131) were significant in favor of the delayed elective approach.

**Conclusions:**

Delayed elective sigmoid resection demonstrates benefit in terms of reduced conversion rates and shortened operative time as opposed to an early approach. Conversely, operative morbidities seem to be unaffected by the timing of surgery. However, a final and robust conclusion based on the included observational cohort studies must be cautiously made. We therefore highly advocate larger randomized controlled trials with homogenous study protocols.

## Introduction

Colonic diverticular disease is one of the most common conditions of the alimentary tract. Especially in western countries, the incidence has been steadily increasing by advanced age as nearly 50% of people older than 60 years have colonic diverticula [1]. Among patients with diverticular disease, approximately 25% develop symptomatic diverticulitis accounting for an annual hospital admission rate of more than 750.000 adults per year across Europe [1–4]. Diverticular disease encompasses a variety of disease stages and thus treatment strategies. While freely perforated diverticulitis with generalized peritonitis is an absolute indication for emergent surgery, the operative approach in complicated or non-complicated acute diverticular disease is widely stage and patient-dependent [5, 6]. Its challenging nature and management regimens are reflected by a portfolio of different national and international guidelines [7]. These guidelines take into account not only the individual disease course but also the varying health care systems of the applying countries. Recently, the latest updated version of the German national guidelines of sigmoid diverticular disease has been published [8]. The recommendation of sigmoid resection in the inflammation-free interval 6 weeks after the initial bout in non-perforated sigmoid diverticulitis is based on a meta-analysis from 2017 with four included non-randomized cohort studies showing comparable clinical outcomes [9]. However, the early elective approach demonstrated a longer operative time and hospital stay with a significantly higher conversion rate [9].

Nevertheless, the exact definition of “early” and “delayed” elective as well as the most appropriate timing of sigmoidectomy seem to be controversial in the literature [10, 11]. This concern arises from cases with a complicated course or early recurrences during the interim period until final surgery, predisposing patients to urgent or emergent resection and an eventful postoperative outcome [5, 12–14]. Furthermore, some more historic considerations propose by analogy to the scenario of the surgical therapy in acute cholecystitis an early elective resection after initial antibiotic therapy as in this stadium post-inflammatory adhesions are less advanced facilitating preparation and resection [15].

Facing these challenges and discrepancies within the surgical community in the management of sigmoid diverticulitis, we seek to provide new insights regarding optimal timing of sigmoidectomy by comprehensively reviewing the current literature on this field and concomitantly performing a meta-analysis. It is intended to systematically compare the perioperative outcome of patients with symptomatic sigmoid diverticular disease undergoing primary laparoscopic sigmoidectomy either in the early elective (EE) or delayed elective (DE) setting.

## Material and methods

The review protocol was registered with the International Prospective Register of Systematic Reviews (PROSPERO Registration Nr. CRD42022307811). The meta-analysis was performed according to the current Preferred Reporting Items for Systematic Reviews and Meta-Analyses (PRISMA) guidelines [16] and the Cochrane Handbook for Systematic Reviews of Interventions [17].

### Literature search

An electronic database search was performed using Pubmed (Medline), Scopus and google scholar, without any time or language restrictions to identify articles comparing the outcome of patients undergoing early elective and delayed sigmoidectomy. The following key search terms were used in combination with the Boolean operators AND or OR: “diverticular disease,” “diverticulitis,” “diverticular,” “surgery,” “time,” “timing,” “early elective,” and “elective.” Furthermore, the reference list of the obtained studies was reviewed to identify potentially relevant citations for the analysis. Two reviewers (S.V. and D.P.) conducted the primary research and independently assessed each abstract and eligible study in terms of relevance for inclusion in the meta-analysis. The last literature research was conducted on the 2nd of March 2022.

### Selection criteria and group definition

Only those studies that analyzed and compared the outcome of patients with non-perforated sigmoid diverticulitis undergoing primary minimally invasive sigmoidectomy either early electively or delayed were included. Early elective sigmoid resection was defined as surgery within 6 weeks from initial hospital admission due to an acute attack while the delayed elective intervention (comparator) was defined as sigmoidectomy after complete symptom amelioration in the inflammation-free interval after 4–6 weeks of the first hospitalization. Publications conducted as randomized controlled trials (RCTs), prospective or retrospective comparative cohort studies applying the Hinchey, Hansen and Stock classification or CDD (classification of diverticular disease) were eligible for analysis. Studies with inconclusive or missing data, performing only cost analysis, primary open approach, purulent or fecal peritonitis, or underlying pathologies other than sigmoid diverticular disease as indication for surgery were excluded. Disagreement or differing conclusions in study selection were either resolved by consensus or consultation of an independent senior surgeon (A.K.).

### Data extraction and outcome measures

Using a self-created electronic data extraction sheet two authors (S.V., D.P.) independently entered all relevant data if fully available from studies meeting eligibility criteria. These include country of origin, year and journal of publication, first author, study design, recruitment period, number of included patients in each group, and their demographic data (age, gender, body mass index [BMI]), American Society of Anesthesiologists (ASA) score, comorbidities, diverticular disease stages, timing of the operative procedure in relation to onset of symptoms, number of previous diverticular attacks and preoperative laboratory results. The primary perioperative endpoint was the conversion rate to open surgery. The secondary operative and postoperative outcome measures analyzed were anastomotic leakage, intraoperative bleeding and blood loss, infected hematoma, intra-abdominal abscess, operative time, peritonitis, postoperative ileus (mechanic/paralytic), surgical site infection, number of ostomies, unplanned surgical re-interventions, trocar hernia, ureteric lesion, and urinary leakage, urinary tract infection, time to first bowel movement after surgery, postoperative pneumonia, postoperative length of hospital stay and the overall mortality. Again disagreement in data extraction was resolved by consensus or re-evaluation of an independent senior surgeon (A.K.).

### Quality assessment

The quality of the included studies was independently assessed by the authors using the ROBINS-I tool [18]. By assessing the risk of bias in non-randomized studies, this instrument covers 7 different domains of bias at 3-time points in each study: pre-intervention (confounding and selection of participants), at intervention (classification of interventions), and post-intervention (biases due to deviations from intended interventions, missing data, measurement of outcomes, and selection of the reported result). With the use of “signaling questions” in each domain, the potential risk of bias could be judged and a final assessment of the overall risk of bias across all domains for every single included study is made. This judgement encompasses the following categories: “Low risk,” “Moderate risk,” “Serious risk,” and “Critical risk” of bias where the low-risk assessment equals the risk of bias in a high-quality randomized trial. In addition, the strength of evidence for the significant primary and secondary outcomes was assessed using the Grading of Recommendations, Assessment, Development, and Evaluation (GRADE) method [19]. Based on the GRADE criteria, including the risk of bias, inconsistency, indirectness of evidence, imprecision, and probability of publication bias, the results were assigned to four levels of evidence (high, moderate, low, and very low) [19, 20].

### Statistical analyses

Statistical analysis was performed using R version 4.1.1 with the package meta [21]. For each outcome of interest, summary estimates of treatment effect were calculated with 95% confidence interval applying a random effects model with the Paule-Mandel (PM) estimator [22] and restricted maximum likelihood (REML) estimator [23] for binary effect size and continuous data, respectively. For dichotomous endpoints, the odds ratio (OR) was chosen as an effect measure. Standardized mean differences (SMDs) were calculated to analyze continuous outcomes.

The level of heterogeneity among the included studies was interpreted as follows after using the Cochrane’s *Q* test (Chi-squared test; Chi^2^) and measuring inconsistency (*I*^2^): 0–30% low heterogeneity, 30–50% moderate heterogeneity, 50–90% substantial heterogeneity [17, 24].

The risk of publication bias was graphically visualized with funnel plots of the natural log of the Odds ratio versus its standard error. Funnel plot symmetry was statistically assessed with the Egger’s test [25] for each outcome mentioned in 5 or more studies.

Meta-regression analysis was performed to explore potential heterogeneity and the impact of country and year of publication and the number of included patients in each study on surgical outcome taking into consideration the differences in medical care between the countries and the ongoing development of minimally invasive colon surgery through the past decades. Subgroup analyses of the significant primary and secondary outcomes were conducted according to study size (≥ median sample size versus < median sample size), study quality (low-moderate versus serious-critical), study design (prospective versus retrospective), and time point of early elective surgery (1–8 days versus 1–42 days).

## Results

**Fig. 1:**
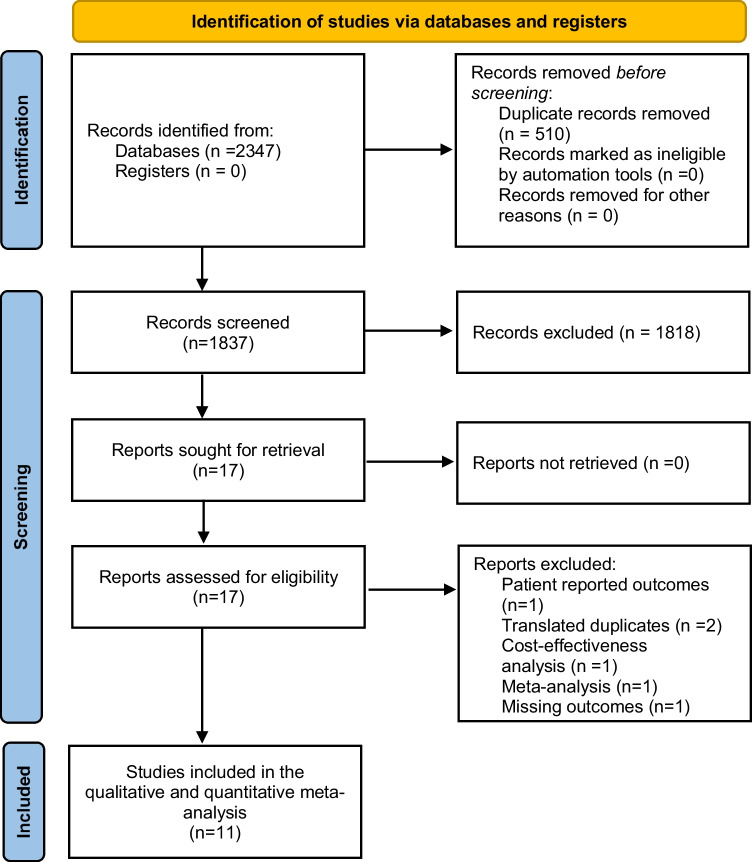
Flow chart diagram of study identification and selection for review analysis

The initial database research with the previously defined keywords identified 2347 potentially relevant abstracts. Of these, 17 full-text articles were assessed for eligibility and finally, 11 studies (8 retrospective and 3 prospective non-randomized cohort studies) comparing the outcome of operative timing in sigmoid diverticular disease were included in the qualitative and quantitative data analysis [26–36]. The Preferred Reporting Items for Systematic Reviews and Meta-Analyses (PRISMA) flow diagram for the literature search is depicted in Fig. 1. From the total of 2096 enrolled patients 828 were assigned to the EE group and 1268 to the DE group.

### Study and patient characteristics

Over an enrollment period from 2003 to 2021, the 11 included studies from six different countries (USA, Egypt, Switzerland, Austria, and Germany) reported their outcomes of primary minimally invasive sigmoidectomy in the early elective and delayed elective setting. All studies excluded patients with free perforation and peritonitis or patients within septic conditions prior to surgery [26–36]. Other exclusion criteria were initial open surgical approach and previous abdominal surgery. Interestingly, two studies also excluded patients in whom an initial conservative therapy regimen had failed, necessitating urgent sigmoidectomy secondarily [27, 28]. Two studies excluded cases with complicated sigmoid diverticulitis [33, 34]. Only one study assessed all stages of an acute and chronic complicated as well as non-complicated sigmoid diverticular disease [36]. Across the included studies four different classification systems of sigmoid diverticular disease (Hinchey, modified Hinchey, Hansen and Stock, and CDD) were applied [8, 37–39]. Two studies did not mention the underlying classification system [26, 33]. Interventional abscess drainage was reported in four studies mainly in the early elective group (8–11%) as opposed to only 4% in patients undergoing delayed resection [30, 32, 33, 36]. Preoperative laboratory findings including inflammatory markers were available in only two studies [30, 36]. With the exception of one study in which single-port sigmoidectomy was performed in 88–100% of cases [35], all remaining studies used the multi-port approach. The study characteristics as well as clinically relevant data are summarized in more detail in Tables 1 and 2.

**Table 1:** Study characteristics

Author	Year	Origin	Study design	Recruitment period	Sample size (*n*)	Exclusion citeria	Diverticular classification system	Diverticulitis stages included	Timing of early elective surgery	Timing of elective surgery	Follow-up period
Natarajan et al. [24]	2004	USA	retrospective	1993–2003	89	extensive previous abdominal surgery, previous colon resection, open surgery	NS	NS	within 30 days of last attack	after 30 days since last attack	30–1095 days
Reissfelder et al. [25]	2006	Germany	prospective, non-randomized	1999–2005	210	free perforation, peritonitis, failure of medical treatment, sepsis, inoperability, previous abdominal surgery	Hansen and Stock, Hinchey	HS IIa, b, III Hinchey I, II	within 5–8 days after antibiotic treatment	after 4–6 weeks since first admission	in hospital
Zingg et al. [26]	2007	Switzerland	retrospective	1997–2005	178	free perforation, peritonitis, failure of medical treatment, open surgery	Hinchey	Hinchey I, II	within 16 days after hospital admission	after ≥ 6 weeks since first admission	30 days
Kirchhoff et al. [27]	2011	Switzerland	prospective, non-randomized	1993–2006	526	free perforation, peritonitis, open surgery, complicated diverticulitis	Hinchey	Hinchey I	within 8 days after admission	after 4–6 weeks since antibiotic treatment	30 days
Hoffmann et al. [28]	2012	Switzerland	retrospective	2005–2009	237	free perforation, open surgery	Hinchey	Hinchey 0, I, II	within 19 days after admission	after 6–8 weeks since last acute attack	in hospital
Zdichavsky et al. [29]	2013	Germany	retrospective	2007–2010	184	free perforation, old patients with positive medical response unfit for surgery, open surgery	Hansen and Stock	HS IIa, b, III	within 10 days after first diverticulitis symptoms	after ≥ 6–8 weeks since last acute attack	in hospital
Warwas et al. [30]	2018	Germany	retrospective	2008–2012	378	free perforation, open surgery	Hansen and Stock	HS 0, I, IIa, b, III	within 8 days after admission	after ≥ 4–6 weeks since last attack subsided	in hospital
Kassir et al. [31]	2019	France	retrospective case-matched	2000–2015	77	free perforation, open surgery, complicated diverticulitis	NS	acute uncomplicated diverticulitis	within 90 days after last attack	after > 13 weeks since last attack	30 days
Abdelkader et al. [32]	2019	Egypt	prospective, non-randomized	2016–2018	47	free perforation, open surgery, complicated diverticulitis, malignancy, ASA > III, psychiatric illness	modified Hinchey	mod. Hinchey Ia	within hospital admission after last attack	after 6–12 weeks since improvement of last attack	in hospital, outpatient
Tschann et al. [33]	2021	Austria	retrospective	2017–2020	37	free perforation, chronic complicated diverticulitis, open surgery	CDD	2 a, b	within 7 days after CT-diagnosis	after 4–6 weeks since CT-based diagnosis	in hospital
Vaghiri et al. [34]	2022	Germany	retrospective	2004–2021	133	free perforation, sepsis, open surgery	CDD	1 b, 2 a, b, 3 a-c	within 42 days after last attack	after > 6 weeks since last diverticulitis attack	in hospital, outpatient

*NS* not stated, *CDD* classification of diverticular disease, *HS* Hansen and Stock

**Table 2:** Patients and therapeutic characteristics

Author	No. of patients	Age (years)	Sex (male /female)	BMI (kg/m^2^)	ASA score	Preoperative CRP	Preoperative leucocytes	No. of previous attacks	Preoperative CT drainage (%)	Complicated diverticulitis (%)	Primary minimally invasive approach (%)	Operative procedure (%)	Stoma creation (%)	No. of performing surgeons
Natarajan et al. [24]	EE 29 DE 60	all patients 52 (24–82)^#^	all patients (52/37)	EE NS DE NS	all patients II (I-III)^#^	EE NS DE NS	EE NS DE NS	all patients 3 (1–10)^#^	EE NS DE NS	EE NS DE NS	EE 100% DE 100%	EE 100% lap. DE 100% lap.	EE 0% DE 0%	7
Reissfelder et al. [25]	EE 116 DE 94	EE 55.4 ± 13.7* DE 56.1 ± 9.8	EE (64/52) DE (49/45)	EE NS DE NS	EE 1.9 ± 0.5* DE 1.8 ± 0.6	EE NS DE NS	EE NS DE NS	EE 1.8 ± 1.7* DE 1.9 ± 1.2	EE NS DE NS	EE NS DE NS	EE 100% DE 100%	EE 100% lap. DE 100% lap.	EE 0% DE 0%	4
Zingg et al. [26]	EE 77 DE 101	EE 60.7 ± 12.5* DE 60.8 ± 11.9	EE NS DE NS	EE 25.5 ± 3.4* DE 26.6 ± 4.1	EE 1.74 (1–3)^#^ DE 1.77 (1–3)	EE NS DE NS	EE NS DE NS	EE NS DE NS	EE NS DE NS	EE 73% DE 13%	EE 100% DE 100%	EE 100% lap. DE 100% lap.	EE 0% DE 0%	NS
Kirchhoff et al. [27]	EE 165 DE 361	all patients 64.2 ± 11.78*	all patients (198/328)	all patients 26.1 ± 4.52*	EE NS DE NS	EE NS DE NS	EE NS DE NS	all patients ≥ 2 episodes	EE NS DE NS	EE 100% DE 100%	EE 100% DE 100%	EE 100% lap. DE 100% lap.	EE 0% DE 0%	13
Hoffmann et al. [28]	EE 81 DE 156	EE 59 ± 15* DE 60 ± 13	EE (44/37) DE (79/77)	EE NS DE NS	EE 2.2 ± 0.6* DE 2.1 ± 0.6	EE 18 (8.3–42)‡ DE 5.0 (4.0–5.0)	EE 8.7 (6.4–11.2)‡ DE 6.9 (6.0–8.1)	EE 0–2 (77%), ≥ 3 (23%) DE 0–2 (49%), ≥ 3 (51%)	EE 11% DE 0%	EE 67% DE 4%	EE 100% DE 100%	EE 94% lap. DE 92% lap.	EE 0% DE 0%	NS
Zdichavsky et al. [29]	EE 91 DE 93	EE 57.6 (32–87)^#^ DE 61.6 (24–80)	EE (49/42) DE (37/56)	EE NS DE NS	EE 1.93† DE 2.00	EE NS DE NS	EE NS DE NS	EE 1 (36%), 2 (17%), > 2 (47%) DE 1 (4%), 2 (12%), > 2 (84%)	EE NS DE NS	EE 100% DE 100%	EE 100% DE 100%	EE 100% lap. DE 100% lap.	EE 2% DE 0%	6
Warwas et al. [30]	EE 100 DE 278	EE 63.6 ± 12.3* DE 61.3 ± 11.5	EE (40/60) DE (94/185)	EE 26.1 ± 3.7* DE 26.3 ± 4.4	EE 2(2)‡ DE 2(2)	EE NS DE NS	EE NS DE NS	EE 2(2)‡ DE 3(3)	EE 0% DE 0%	EE 79% DE 83%	EE 100% DE 100%	EE 95% lap. DE 97% lap.	EE 1% DE 0.4%	11
Kassir et al. [31]	EE 39 DE 38	EE 54.6 (24–88)^-^ DE 55.5 (28–84)	EE (21/18) DE (18/20)	EE 26 (18–38)^-^ DE 25 (17–41)	EE I/II (87%), III/IV (13%) DE I/II (92%), III/IV (18%)	EE NS DE NS	EE NS DE NS	EE 1.4† DE 1.6	EE 0% DE 0%	EE 0% DE 0%	EE 100% DE 100%	EE 100% lap. DE 100% lap.	EE 5% DE 0%	NS
Abdelkader et al. [32]	EE 25 DE 22	EE 60.38 ± 8.25* DE 61.41 ± 6.92	EE (11/14) DE (20/12)	EE 29.59 ± 3.01* DE 30.11 ± 2.42	EE 1.65 ± 0.68* DE 1.81 ± 0.59	EE NS DE NS	EE NS DE NS	EE NS DE NS	EE NS DE NS	EE 0% DE 0%	EE 100% DE 100%	EE 92% lap. DE 100% lap.	EE 8% DE 0%	NS
Tschann et al. [33]	EE 17 DE 20	EE 56.2 ± 11* DE 59.9 ± 12.2	EE (11/6) DE (9/11)	EE 25.2 ± 2* DE 25.4 ± 4.2	EE 2.0 ± 0.6* DE 2.2 ± 0.7	EE NS DE NS	EE NS DE NS	EE 0 DE 0	EE NS DE NS	EE 100% DE 100%	EE 100% DE 100%	EE 88% single-port DE 100% single-port	EE 0% DE 0%	NS
Vaghiri et al. [34]	EE 88 DE 45	EE 56 ± 12.3^Ɨ^ DE 53 ± 13.1	EE (42/46) DE (27/18)	EE 26.6 ± 5.4^Ɨ^ DE 25.9 ± 3.9	EE 2 ± 0.68^Ɨ^ DE 2 ± 0.79	EE 4.6 ± 7.6^Ɨ^ DE 0.3 ± 3.6	EE 10.7 ± 5.2^Ɨ^ DE 7.5 ± 2.6^Ɨ^	EE 2 ± 1.2^Ɨ^ DE 2 ± 1.6	EE 8% DE 4%	EE 66% DE 58%	EE 100% DE 100%	EE 100% lap. DE 100% lap	EE 6% DE 0%	NS

*EE* early elective, *DE* delayed elective, *SD* standard deviation, *IQR* interquartile range, *BMI* body mass index, *ASA* American Society of Anesthesiology, *CRP* C-reactive protein, *lap*. laparoscopic, *NS* not stated, *mean ± *SD*, ^#^mean (range), ‡median (IQR), †mean, ^-^ median (range), ^Ɨ^median ± *SD*

### Study quality and risk of bias

**Fig. 2:**
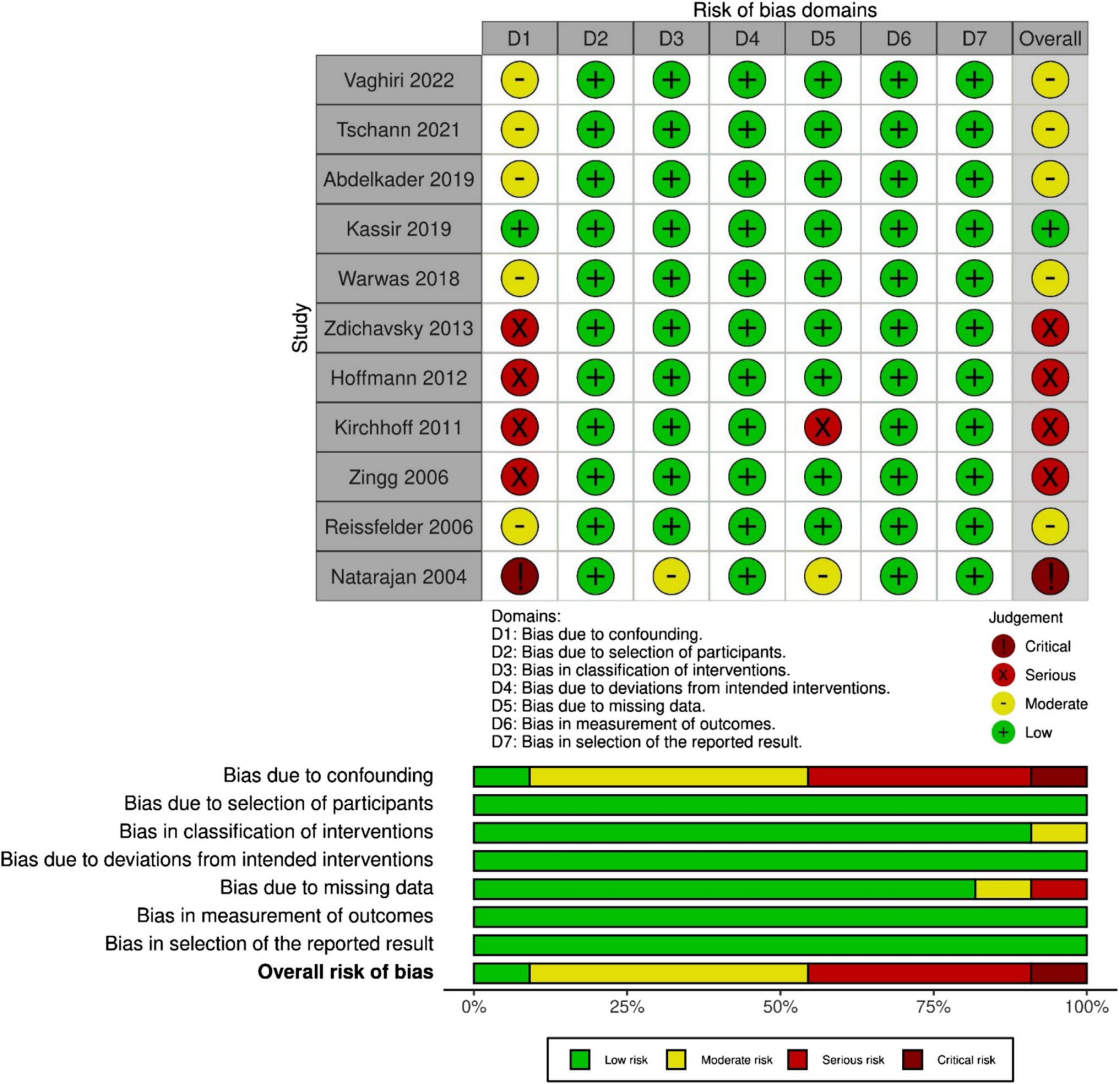
Risk of bias summary and graphical visualization of the included studies based on ROBINS-I tool

The risk of bias (Fig. 2) in the majority of included studies was moderate to serious with the exception of one study [33] demonstrating low bias risk according to the Robins-I tool [18]. However, the main limiting factor concerning bias was the non-randomized conception of all studies. Based on the GRADE method, the level of evidence for the significant primary and secondary outcomes was rated as low or very low (Table 3).

**Table 3:** The GRADE Certainty assessment for the significant primary and secondary outcomes

Outcomes	No. included of studies	No. of included patients		SMD/OR [95% CI]	Quality assessment					Quality
		EE	DE		Risk of bias	Inconsistency	Indirectness	Imprecision	Publication bias	
Primary										
Conversion rate	11 [26–36]	111/828	70/1268	OR 2.48 (1.5427–4.0019)	Serious (− 1)	No inconsistency	No indirectness	No imprecision	No bias	Low
Secondary										
Operative time	9 [27, 28, 30–36]	634	847	SMD 0.14 (0.0020–0.2701)	Serious (− 1)	Serious (− 1)	No indirectness	No imprecision	No bias	Very low
Bowel movement	2 [27, 33]	155	132	SMD 0.57 (0.1202–1.0233)	Serious (− 1)	Very serious (− 2)	No indirectness	Serious (− 1)	Unclear	Very low

*DE* delayed elective, *EE* early elective, *OR* odds ratio, *SMD* standardized mean difference

^a^Risk of bias assessed using the ROBINS-I tool

^b^Publication bias was assessed by Egger’s test

### Primary outcome analysis

#### Conversion rate to open surgery

**Fig. 3:**
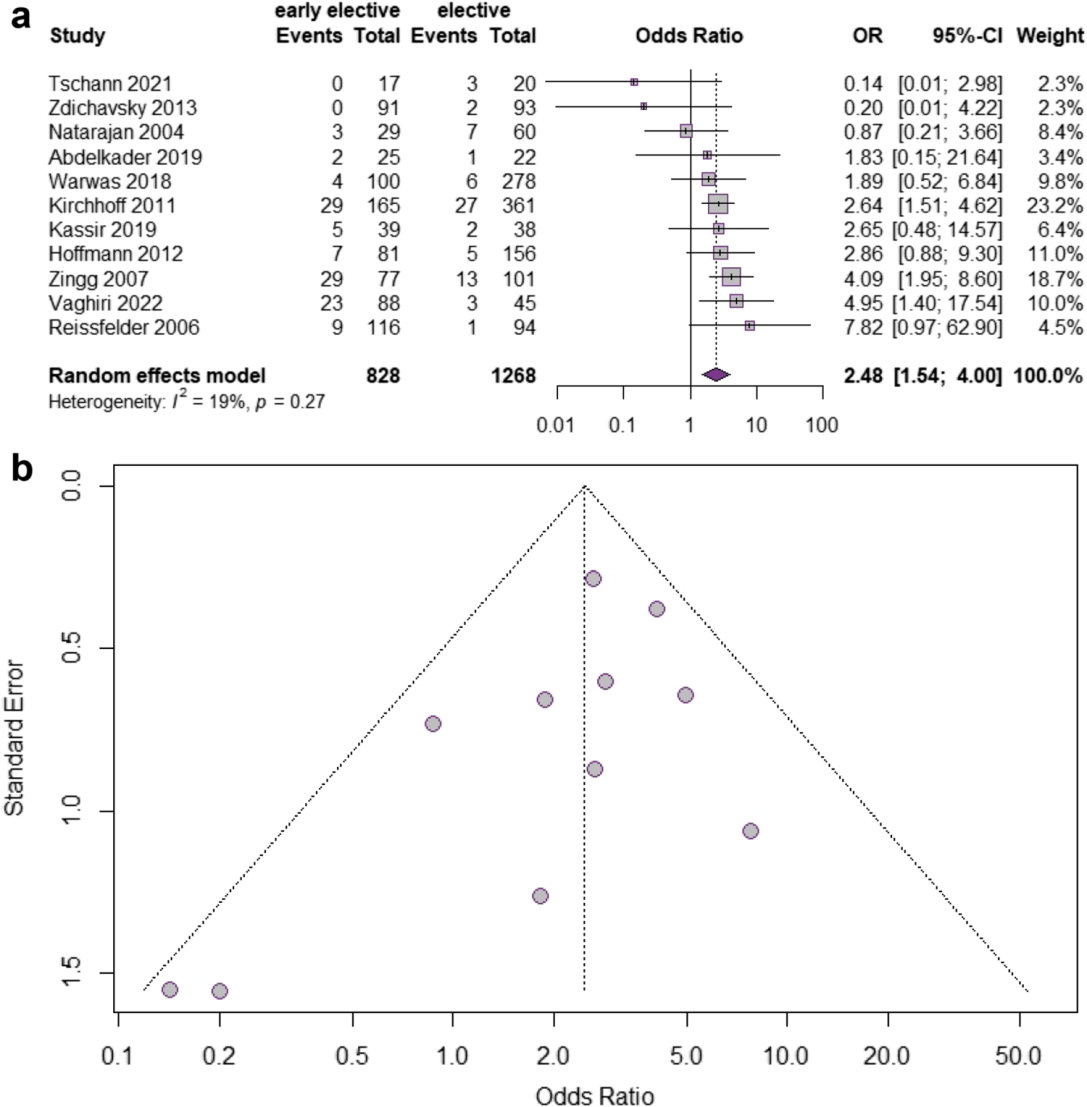
Meta-analysis comparing the conversion rate in early elective versus delayed elective sigmoid resection. **a** Forest plot reflects the individual and pooled OR with 95% CI for the relationship between early elective or delayed elective sigmoidectomy and conversion rate to open surgery. **b** Funnel plot of the included studies for the conversion rate to laparotomy. The *Y*-axis represents the standard error (SE), and the *x*-axis represents the study’s result

Conversion rate as the primary endpoint was reported for all 2096 patients in the 11 included studies without exception [26–36] (Fig. 3a). Strikingly, conversion rates were significantly higher in the EE group in comparison to the DE cohort irrespective of the disease stage (OR 2.48, 95% CI 1.5427–4.0019, *p* = 0.0002). Importantly, heterogeneity was low (*I*^2^ = 19%, Chi^2^-test: *p* = 0.27). Egger’s test (*p* = 0.13) and funnel plot (Fig. 3b) showed no evidence of publication bias.

### Secondary outcome analysis

#### Statistically significant secondary outcomes

##### *Operative Time*

**Fig. 4:**
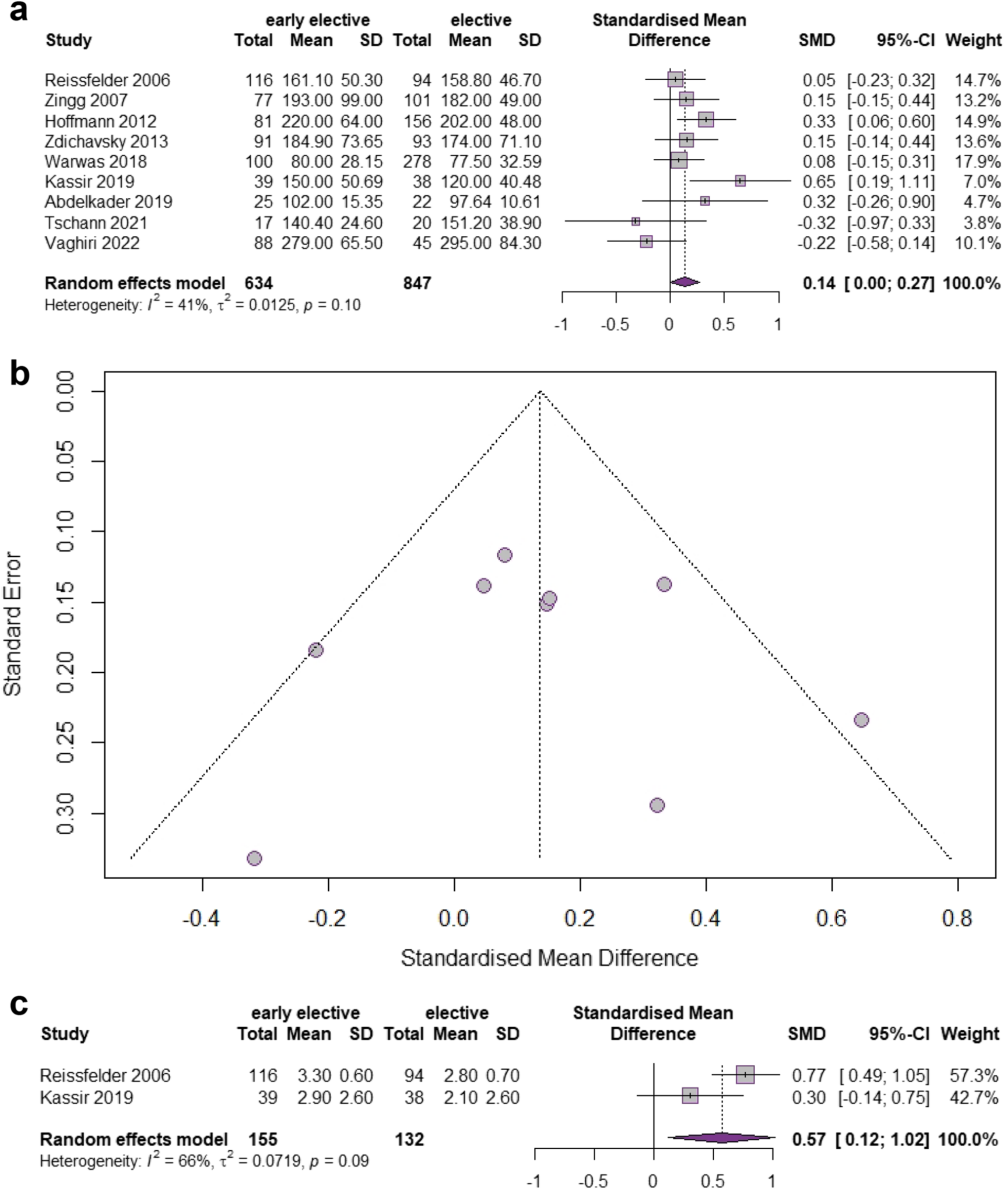
Meta-analysis comparing **a** and **b** the operative time and **c** time in days to first bowel movement in early elective versus delayed elective sigmoid resection. **a** Forest plot reflects the individual and pooled SMD with 95% CI for the relationship between early elective or delayed elective sigmoidectomy and duration of surgery. **b** Funnel plot of the included studies for operative time. The Y-axis represents the standard error (SE), and the x-axis represents the study’s result. **c** Forest plot reflects the individual and pooled SMD with 95% CI for the relationship between early elective or delayed elective sigmoidectomy and time in days to first bowel movement after surgery

The reported duration of the operative procedure was significantly shorter in DE sigmoidectomy in comparison to the EE resection in the 9 included studies [27, 28, 30–36] with a total of 1481 patients (SMD 0.14, 95% CI 0.0020–0.2701, *p* = 0.0466) (Fig. 4a). The heterogeneity level was moderate (*I*^2^ = 41%, Chi2-test: *p* = 0.10). Funnel plot (Fig. 4b) was symmetric (Egger’s test: *p* = 0.97).

##### *Bowel movement*

Two studies encompassing 287 patients investigated the time in days to first bowel movement after surgery [27, 33] (Fig. 4c). In patients with a delayed elective resection bowel movement was restored more quickly after surgery in comparison to the early elective group (SMD 0.57, 95% CI 0.1202–1.0233, *p* = 0.0131). Of note the level of heterogeneity was considerably high (*I*^2^ = 66%, *p* = 0.09). Due to the small number of studies, the test for publication bias and the preparation of a funnel plot were omitted.

#### Statistically non-significant secondary variables

Non-significant differences between EE and DE sigmoidectomy became evident for the following outcomes: anastomotic-leakage, bleeding, blood loss, infected hematoma, intra-abdominal abscess, peritonitis, postoperative ileus, surgical site infection, stoma creation, revision surgery, trocar hernia, ureter lesion, urinary tract infection, postoperative pneumonia, postoperative length of hospital stay, and the mortality (Table 4).

**Table 4:** Non-significant secondary outcomes

Secondary outcomes	No. of included studies	No. of included patients	SMD/OR [95% CI]	*P*-value	Heterogeneity level		Publication bias
					*I*^2^ (%)	*P*-value	*P*-value
Anastomotic-leakage	10 [24–26, 28–34]	1567	OR 1.25 [0.6255–2.5146]	0.5234	0	0.59	0.88
Bleeding	8 [25, 26, 28–30, 32–34]	1404	OR 1.30 [0.6443–2.6167]	0.4651	0	0.91	0.42
Blood loss	2 [26, 32]	225	SMD 0.85 [− 0.3901–2.0804]	0.1799	92	< 0.01	NA
Infected hematoma	3 [25, 33, 34]	380	OR 0.31 [ 0.0318–3.1039]	0.3219	0	0.89	NA
Intra-abdominal abscess	5 [26, 28, 29, 31, 34]	809	OR 0.99 [0.3768–2.6108]	0.9867	0	0.61	0.39
Peritonitis	2 [25, 29]	394	OR 1.72 [0.0816–36.0340]	0.7285	49	0.16	NA
Postoperative ileus	6 [25, 26, 28, 29, 32, 34]	989	OR 1.18 [0.4964–2.8229]	0.7035	0	0.97	0.19
Surgical site infection	8 [25, 26, 28–32, 34]	1444	OR 1.58 [0.9214–2.6990]	0.0966	10	0.35	0.79
Stoma creation	9 [25, 26, 28–34]	1481	OR 3.19 [0.8621–11.7823]	0.0823	0	0 .88	0.25
Revision surgery	6 [29–34]	856	OR 1.02 [0.5194–1.9895]	0.9618	0	0.63	0.65
Trocar hernia	3 [29, 31, 34]	394	OR 1.16 [0.1805–7.4490]	0.8762	0	0.63	NA
Ureter lesion	7 [25, 26, 30–34]	1060	OR 0.91 [0.2591–3.2186]	0.8876	0	0.90	0.93
Urinary tract infection	2 [29, 31]	261	OR 0.65 [0.0597–6.9599]	0.7175	36	0.21	NA
Postoperative Pneumonia	3 [29, 31, 34]	394	OR 2.91 [0.3156–26.8636]	0.3458	0	0.60	NA
Postoperative length of hospital stay	6 [28–30, 32–34]	1016	SMD − 0.02 [− 0.1517–0.1111]	0.7618	36	0.17	0.31
Mortality	9 [25, 26, 28–34]	1481	OR 0.99 [0.1023–9.6700]	0.9964	0	0.70	NA*

*OR* odds ratio, *SMD* standardized mean difference, *NA* not applicable, *only two events

##### *Meta-regression analyses*

**Fig. 5:**
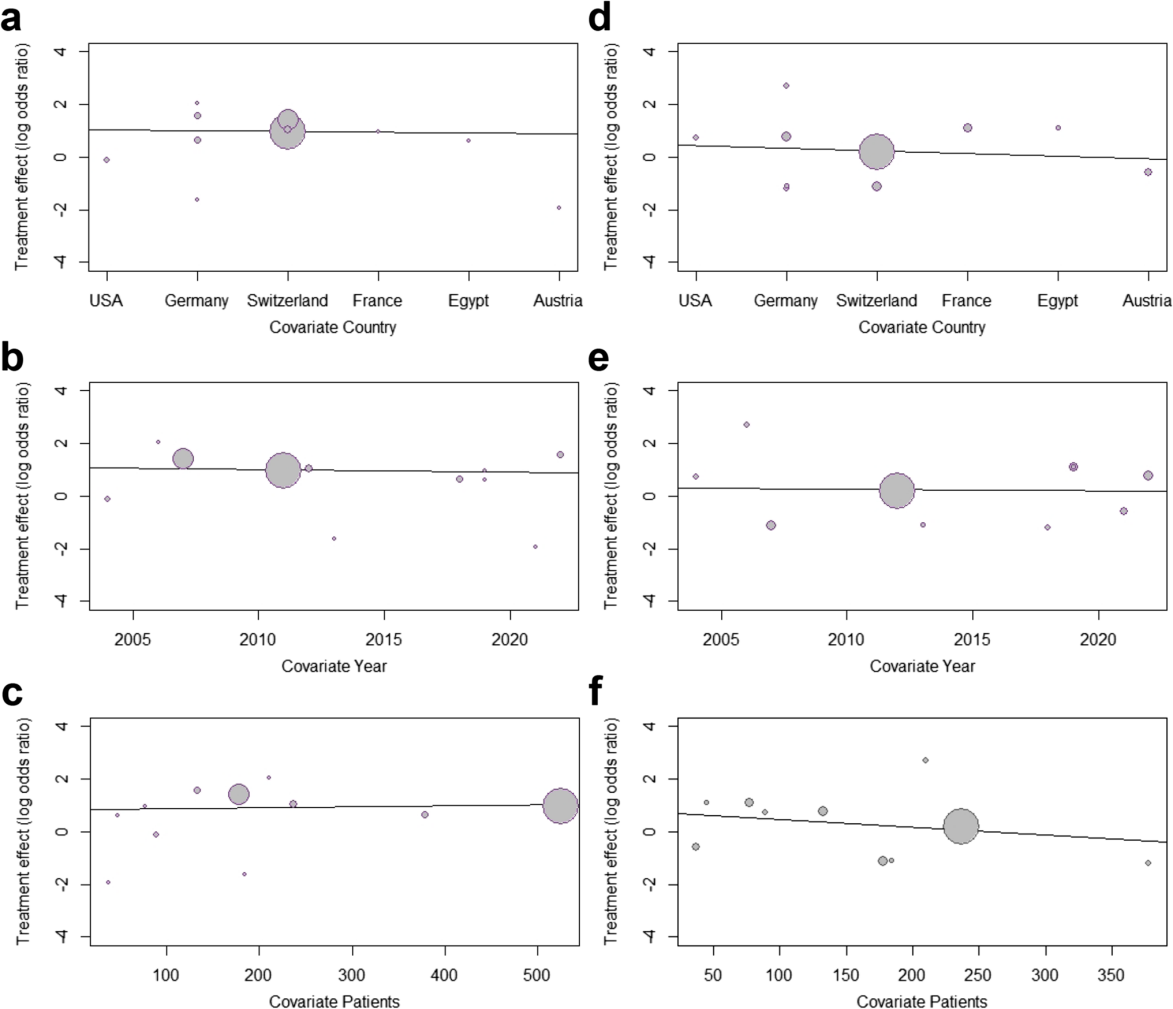
Scatter plot of the relationship between **a** and **d** country of origin, **b** and **e** publication year, and **c** and **f** study size and log odds ratio for **a–c** conversion rate, and **d–f** anastomotic-leakage

The meta-regression analyses investigated the potential effects of three clinical confounders (country of origin, year of publication, and sample size of included studies) on the conversion rate to open surgery and anastomotic-leakage in relation to timing of sigmoidectomy (Fig. 5).

#### Conversion rate to open surgery

Random-effects meta-regression revealed no statistical significance evidencing potential association between country of origin, publication date or sample size, and the conversion rate (*p* > 0.05) (Fig. 5a-c).

#### Anastomotic-leakage

Meta-regression analyses revealed no statistically significant association between country of origin, publication date, or sample size, and the rate of anastomotic-leakage (*p* > 0.05) (Fig. 5d-f).

##### *Subgroup analyses*

A subgroup analysis was performed to determine the consistency of the reported statistically significant primary and secondary outcomes and to clarify the effects of study size, study bias, study design, and time interval of early elective sigmoidectomy on the results (Table 5). As the secondary outcome variable bowel movement was only investigated by two studies, we could not perform a subgroup analysis for this factor, so it was only analyzed for conversion rate and operative time. Studies with a larger patient population (≥ 178 cases) [27–32] showed a stronger association between the timing of surgery and the two outcome variables of interest. Interestingly, however, we found no significant difference for the conversion to open surgery in the group of studies that defined early elective sigmoidectomy within the first 1–8 days after hospitalization [27, 29, 32, 35]. In contrast, the results of the subgroup analysis on operative time must be interpreted with great caution due to heterogeneity.

**Table 5:** Subgroup analysis of significant primary and secondary outcomes

	Conversion rate						Operative time					
Subgroup	No. of included studies	No. of included patients	OR [95% CI]	*P*-value	*I*^2^ (%)	Chi^2^ (*P*-value)	No. of included studies	No. of included patients	SMD [95% CI]	*P*-value	*I*^2^ (%)	Chi^2^ (*P*-value)
Study size												
< 178 cases	5 [26, 33–36]	383	OR 1.73 [0.6282–4.7723]	0.2886	36	0.1798	4 [33–36]	294	SMD 0.11 [− 0.3413–0.5689]	0.6241	72	0.0139
≥ 178 cases	6 [27–32]	1713	OR 2.89 [1.9047–4.3940]	< 0.0001	4	0.3939	5 [27, 28, 30–32]	1187	SMD 0.15 [0.0261–0.2657]	0.0170	0	0.6153
Study bias												
Low-moderate	6 [27, 32–36]	882	OR 2.63 [1.1657–5.9127]	0.0198	15	0.3183	6 [27, 32–36]	882	SMD 0.09 [− 0.1482–0.3261]	0.4623	53	0.0581
High-critical	5 [26, 28–31]	1214	OR 2.24 [1.0847–4.6379]	0.0293	37	0.1717	3 [28, 30, 31]	599	SMD 0.22 [0.0521–0.3809]	0.0099	0	0.5701
Study design												
Prospective	3 [27, 29, 34]	783	OR 2.78 [1.6390–4.7214]	0.0002	0	0.5796	2 [27, 34]	257	SMD 0.09 [− 0.1492–0.3429]	0.4402	0	0.3997
Retrospective	8 [26, 28, 30–33, 35, 36]	1313	OR 2.07 [1.0246–4.1990]	0.0426	37	0.1325	7 [28, 30–33, 35, 36]	1224	SMD 0.14 [− 0.0386–0.3176]	0.1248	53	0.0490
Time interval “early elective”												
1–8 days	4 [27, 29, 32, 35]	1151	OR 2.07 [0.5839–7.334]	0.2599	37	0.1887	3 [27, 32, 35]	625	SMD 0.04 [− 0.1291–0.2090]	0.6430	0	0.5265
1–42 days	4 [28, 30, 31, 36]	732	OR 3.06 [1.2026–7.8073]	0.0189	24	0.2666	4 [28, 30, 31, 36]	732	SMD 0.12 [− 0.0836–0.3325]	0.2411	48	0.1223

*OR* odds ratio, *SMD* standardized mean difference

## Discussion

We conducted a systematic review and meta-analysis including 11 studies with an overall moderate to high risk of bias and mostly low heterogeneity level for the outcomes of interest except operative time, peritonitis, urinary tract infection, postoperative hospital stay (moderate heterogeneity), intraoperative blood loss and postoperative bowel movement (high heterogeneity level). The results demonstrate a higher conversion rate and longer operative time if sigmoidectomy is performed in the early elective period after an acute attack. Other variables including operative morbidities (anastomotic-leakage, intraoperative blood loss, and bleeding, intra-abdominal abscess, infected hematoma, ureteric lesion, postoperative ileus, surgical site infection, peritonitis, stoma creation, revision surgery, and trocar hernia), postoperative urinary tract infection, postoperative pneumonia, length of hospital stay and mortality seem not to be influenced by the time point of sigmoid resection. Nevertheless, we could demonstrate that patients undergoing delayed elective sigmoidectomy have a faster return of regular bowel movement after surgery in comparison to the early elective group although displaying a considerably high heterogeneity level among the two included studies [27, 33]. Furthermore, the meta-regression analyses revealed no statistically significant association between study-specific data (year of publication, country of origin, or number of included patients) and the conversion or anastomotic-leakage rates, suggesting equally distributed surgical standards across the contributing countries.

Sigmoid diverticular disease is a widespread and common gastrointestinal condition accounting for a high annual hospitalization rate and striking socio-economic costs to western countries’ health care systems [40–42]. The therapeutic strategies vary depending on disease stage, disease burden, and the individual risk factors [6, 14]. While uncomplicated cases are treated mostly non-operatively with or without antibiotics considering the low risk of recurrence and complications [43–45], acute or chronic diverticular disease accompanied by abscess formation, fistula, stenosis or stricture will mostly require resection after symptom relief either early electively from index hospitalization to 4–6 weeks after the acute attack or in the inflammation-free interval after 6 weeks [5, 8, 46, 47]. Patients with frank perforation and septic conditions need emergent sigmoidectomy at initial presentation [6]. In the past, the surgical approach and timing of resection were mainly based on the preference of the involved surgeons. Technical refinements in minimally invasive colorectal surgery have evolved over the past years. Nowadays laparoscopic sigmoid resection is a well-standardized and established method in the treatment of diverticular disease even in complicated cases [48, 49]. This procedure demonstrated superior short-term results regarding enhanced recovery, shorter hospitalization time, improved quality of life, and reduced morbidities with similar health care costs compared to the open approach in numerous randomized and non-randomized studies [50–55]. For elective laparoscopic sigmoidectomy overall mortality and morbidity rates of 1% and 9–10%, respectively, are reported while conversion to laparotomy is observed in approximately 9–20% [50, 56]. Consistent with this data, the overall mortality rate in this study was approximately 0.1% across both groups. A conversion rate of 8.6% was recorded.

The recommendation of sigmoidectomy during the elective interval of 4–6 weeks after cessation of symptoms has become the standard and has been incorporated in recent guidelines of diverticular disease although randomized controlled trials addressing this question are still missing [8]. Khan et al. [9] were the first to conduct a meta-analysis investigating the outcome of timing in surgical resection for diverticular disease. Their meta-analysis included four observational studies with a total of 1046 patients undergoing both, open and laparoscopic sigmoidectomy. While no difference in surgical site infection, intra-abdominal abscess formation, anastomotic leakage, 30-day mortality, postoperative ileus, postoperative bleeding, ureteric injury, and overall morbidity became evident, early elective surgery was associated with a prolonged operative time, higher conversion rates and longer hospital stay. Our results are in line with this observation except no statistically significant difference in the length of hospital stay. However, the meta-analysis by Khan et al. [9] displays some weaknesses: (1) three eligible studies [26, 29, 31] were not included in the meta-analysis; (2) data extraction and analysis were erroneous for certain outcome variables (conversion rate, length of hospital stay); (3) the analysis of the length of hospital stay performed by Khan and colleagues [9] is composed both of studies that investigated the total hospital stay [28] or described only the postoperative stay [30]. In addition, Bachmann and co-workers [12] did not precisely define whether it was the entire hospital stay or the postoperative length of stay; (4) Furthermore, the study by Bachmann et al. [12] does not specify how many patients in each group (EE versus DE surgery) underwent primary laparoscopic sigmoidectomy or open sigmoid resection. Thus, the conclusion regarding conversion rates by Khan and co-workers [9] is somewhat misleading, and we therefore excluded the study by Bachmann et al. [12] from our analysis. In our recently published work [36] by applying the CDD classification we could demonstrate that besides timing of sigmoid resection the disease stage influences the conversion rates in laparoscopic sigmoidectomy for diverticular disease. The reason for conversion in the early elective resection strategy is mainly due to ongoing inflammation of the tissue with surrounding adhesions making laparoscopic dissection rather difficult and challenging in the short time period after hospitalization [28, 33, 36, 57]. Therefore advanced and complicated disease stages are more likely leading to higher conversion rates in the early elective phase. Notably, the distribution of sigmoid diverticular disease stages throughout the analyzed studies is not homogenous. While some authors [33, 34] exclude cases with complicated disease stages, others [28, 30–32, 36] mixed cases with chronic or complicated and non-complicated sigmoid diverticulitis.

The hereby presented results must be cautiously interpreted as all the included and analyzed studies are non-randomized with the limited methodical quality attributed to observational cohort studies. Interestingly, no uniform definition of the early elective and delayed elective intervention period in relation to the disease onset exists. Interpretation of “early elective” resection in the included studies ranges from 1–8 days to 90 days since index hospitalization while “delayed elective” surgery is performed from 30 days to 13 weeks after initial presentation. The results of our subgroup analysis suggest that the conversion rate to the open procedure is lower than at a later time point, especially during the first 8 days. This could possibly be due to the fact that the pathological inflammatory reaction during the first 1–8 days of sigmoid diverticulitis is less pronounced than previously assumed. Most importantly the decision towards early elective or delayed surgical approach is subject to confounding as there is no randomization in the included studies and the group affiliation is potentially driven by the surgeon’s personal preference. Possible selection bias could be considering patients with disease progression under conservative therapy or a more severe disease course for an early elective sigmoidectomy. Higher conversion rates and prolonged operative time in patients undergoing early elective sigmoidectomy with no differences in length of postoperative hospital stay and morbidities in comparison to the delayed resection could potentially call for some considerations of the total treatment cost-effectiveness, including the risk of an eventful recurrent disease course during the waiting period and days off of work, as early elective sigmoidectomy prevents a second hospitalization outweighing the mentioned benefits of delayed sigmoid resection. Against this background and given the very low quality of evidence, the results presented significantly limit the strength of the recommendation in daily clinical practice. In particular, the question of the best time for minimally invasive resection after symptoms have subsided in an era of selective, patient-centered approaches remains unanswered.

## Conclusion

Timing of sigmoid resection in diverticular disease is still a matter of debate. Delayed elective sigmoidectomy consistently demonstrates lower conversion rates and shortened operative time while no significant differences in perioperative morbidities, length of hospital stay, or mortality in comparison to the early elective approach within 6 weeks after the attack became evident. However, definite conclusions based on the available literature are difficult to draw as randomized controlled studies are scarce. We therefore advocate multicenter RCTs with homogenous disease stages, classification systems, and comparable study protocols (especially with respect to complicated subtypes of diverticular disease and a consistent definition of early and delayed elective resection) targeting this relevant subject with striking socio-economic impact on our health care system.
